# Effectiveness of icosapent ethyl on first and total cardiovascular events in patients with metabolic syndrome, but without diabetes: REDUCE-IT MetSyn

**DOI:** 10.1093/ehjopen/oead114

**Published:** 2023-11-12

**Authors:** Michael Miller, Deepak L Bhatt, Eliot A Brinton, Terry A Jacobson, Philippe Gabriel Steg, Armando Lira Pineda, Steven B Ketchum, Ralph T Doyle, Jean-Claude Tardif, Christie M Ballantyne

**Affiliations:** Department of Medicine, Corporal Michael J. Crescenz Veterans Affairs Medical Center and Hospital of the University of Pennsylvania, 3900 Woodland Avenue, Philadelphia, PA 19104-4551, USA; Mount Sinai Fuster Heart Hospital, Icahn School of Medicine at Mount Sinai, New York, NY, USA; Utah Lipid Center, Salt Lake City, UT, USA; Lipid Clinic and Cardiovascular Risk Reduction Program, Department of Medicine, Emory University School of Medicine, Atlanta, GA, USA; Université de Paris, FACT (French Alliance for Cardiovascular Trials), Assistance Publique–Hôpitaux de Paris, Hôpital Bichat, INSERM Unité 1148, Paris, France; Amarin Pharma, Inc. (Amarin), Bridgewater, NJ, USA; Amarin Pharma, Inc. (Amarin), Bridgewater, NJ, USA; Amarin Pharma, Inc. (Amarin), Bridgewater, NJ, USA; Montreal Heart Institute, Université de Montréal, Montreal, Canada; Department of Medicine, Baylor College of Medicine, Texas Heart Institute, Houston, TX, USA

**Keywords:** Metabolic syndrome, Diabetes, Hypertension, Hypertriglyceridaemia, HDL-C, Icosapent ethyl, Obesity

## Abstract

**Aims:**

Metabolic syndrome (MetSyn) is associated with high risk of cardiovascular (CV) events, irrespective of statin therapy. In the overall REDUCE-IT study of statin-treated patients, icosapent ethyl (IPE) reduced the risk of the primary composite endpoint (CV death, non-fatal myocardial infarction, non-fatal stroke, coronary revascularization, or unstable angina requiring hospitalization) and the key secondary composite endpoint (CV death, non-fatal myocardial infarction, or non-fatal stroke).

**Methods and results:**

REDUCE-IT was an international, double-blind trial that randomized 8179 high CV risk statin-treated patients with controlled LDL cholesterol and elevated triglycerides to IPE 4 g/day or placebo. The current study evaluated the pre-specified patient subgroup with a history of MetSyn, but without diabetes at baseline. Among patients with MetSyn but without diabetes at baseline (*n* = 2866), the majority (99.8%) of this subgroup was secondary prevention patients. Icosapent ethyl use was associated with a 29% relative risk reduction for the first occurrence of the primary composite endpoint [hazard ratio: 0.71; 95% confidence interval (CI): 0.59–0.84; *P* < 0.0001, absolute risk reduction (ARR) = 5.9%; number needed to treat = 17] and a 41% reduction in total (first plus subsequent) events [rate ratio: 0.59; (95% CI: 0.48–0.72); *P* < 0.0001] compared with placebo. The risk for the key secondary composite endpoint was reduced by 20% (*P* = 0.05) and a 27% reduction in fatal/non-fatal MI (*P* = 0.03), 47% reduction in urgent/emergent revascularization (*P* < 0.0001), and 58% reduction in hospitalization for unstable angina (*P* < 0.0001). Non-statistically significant reductions were observed in cardiac arrest (44%) and sudden cardiac death (34%).

**Conclusion:**

In statin-treated patients with a history of MetSyn, IPE significantly reduced the risk of first and total CV events in REDUCE-IT. The large relative and ARRs observed supports IPE as a potential therapeutic consideration for patients with MetSyn at high CV risk.

**Registration** REDUCE-IT ClinicalTrials.gov number: NCT01492361

## Graphical Abstract

**Graphical Abstract:**
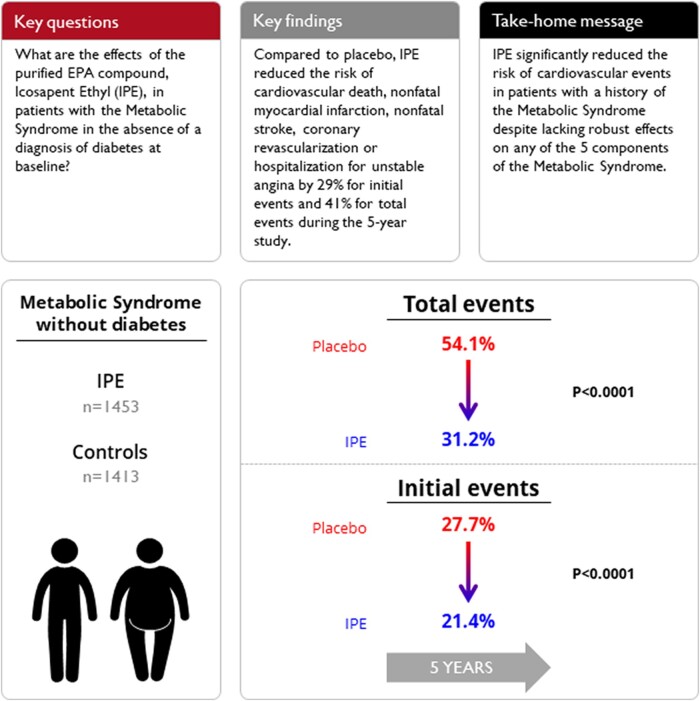

## Introduction

More than one of every three adult Americans have the metabolic syndrome (MetSyn),^1^ a cluster of three or more of the following five risk factors: (i) waist circumference ≥40 inches (102 cm) in men and ≥35 inches (88 cm) in women, (ii) blood pressure ≥130/85 mmHg, (iii) fasting glucose ≥100 mg/dL, (iv) triglycerides (TGs) ≥150 mg/dL, and (v) HDL-C <40 mg/dL in men and <50 mg/dL in women.^2^ Metabolic syndrome is not only a recognized risk factor for incident diabetes but also raises the risk of adverse cardiovascular disease (CVD) outcomes [e.g. myocardial infarction, stroke, and cardiovascular (CV) mortality] by at least two-fold, even in the absence of diabetes.^3^ In recent years, MetSyn has also been linked to a variety of pathologic phenotypes including heart failure^4^ and renal insufficiency.^5^ While intensive lifestyle changes that result in significant weight loss invariably improve the risk-factor profile associated with MetSyn,^6^ there are surprisingly few data evaluating either lifestyle and/or pharmacologic therapy aimed at reducing CVD risk in patients with MetSyn. Recently, icosapent ethyl (IPE), a purified formulation of eicosapentaenoic acid (EPA), was demonstrated in the Reduction of Cardiovascular Events with Icosapent Ethyl Trial (REDUCE-IT) to reduce CVD events in men and women with hypertriglyceridaemia (HTG) and established CVD or elevated CVD risk.^7^ However, evaluation of IPE in patients with MetSyn was not a primary analysis in the REDUCE-IT study. Consequently, the rationale for the current study, of Metsyn as a pre-specified subgroup analysis in REDUCE-IT, was two-fold: (i) examine the extent to which IPE may be beneficial in patients with MetSyn who did not have diabetes at baseline and (ii) determine whether IPE had any impact on the risk of new-onset diabetes in patients with MetSyn.

## Methods

REDUCE-IT was a randomized double-blind, placebo-controlled Phase 3b trial of statin-treated patients with HTG [150–499 mg/dL (1.69–5.63 mmol/L)] and LDL cholesterol, 41–100 mg/dL (1.06–2.59 mmol/L), assigned to either IPE or placebo (4 g daily) after dietary counselling. The study design and results have been reported in the past.^6,7^ Efficacy analyses were conducted using the intention-to-treat (ITT) approach. All study sites were approved by the respective ethics committee or institutional review board. A composite of CV death, non-fatal myocardial infarction (MI), non-fatal stroke, coronary revascularization, or unstable angina resulting in hospitalization represented the primary endpoint, whereas CV death, non-fatal MI, or non-fatal stroke comprised the key secondary composite endpoint. Other endpoints included CV death or non-fatal MI, fatal or non-fatal MI, urgent or emergent revascularization, CV death, hospitalization for unstable angina, fatal or non-fatal stroke, the combination of total mortality, non-fatal MI and non-fatal stroke, total mortality, cardiac arrest, and sudden cardiac death. The current analysis evaluated hazard ratios (HRs) and 95% confidence intervals (CIs) in patients distinguished by a history of MetSyn as characterized by three or more criteria noted above but without diabetes at baseline. Diabetes was defined by a history of elevated blood glucose [fasting levels exceeding 125 mg/dL (6.9 mmol/L)] requiring medication with new-onset diabetes similarly defined during interval history and/or follow-up visits.

### Statistical analysis

Baseline characteristics were examined between treatment groups with categorical and continuous variables compared using the *χ*^2^ test and Wilcoxon rank-sum test, respectively. The time to the initial occurrences of both primary and secondary composite endpoints was assessed by Kaplan–Meier analysis based upon CV risk category, geographic region, and prescribed use of ezetimibe at baseline. Initial and recurrent (total) events were analysed using a negative binomial regression model as previously conducted.^8^ Hazard ratios and 95% CIs were generated from a corresponding stratified Cox proportional-hazards regression model. *P*-values presented are nominal and exploratory with no adjustment for multiple comparisons. Statistical analyses were performed using SAS software, version 9.4 (SAS Institute, Cary, NC, USA).

## Results

### Baseline characteristics

Of the 8179 patients enrolled in REDUCE-IT, 35% (*n* = 2866) of the ITT population had MetSyn (≥3 factors) without diabetes at baseline. The majority (99.8%) of this subgroup was secondary prevention patients. As shown in *Table 1*, baseline characteristics included the median age of 62 years with women comprising 19.9% (*n* = 569). In addition to HTG (TG ≥ 150 mg/dL), which served as an entry criterion for REDUCE-IT, the other MetSyn factors with prevalence were increased waist circumference (men, 66.3%; women 87.9%), hypertension (82%), impaired fasting glucose (100–125 mg/dL; 57.9%), and low HDL-C (men, 60.3%; women, 74.5%), with assignment to IPE associated with higher prevalence of low HDL-C for women (*P* = 0.01). Patients taking medications commonly prescribed with MetSyn (*Table 2*) did not demonstrate differences at baseline whether assigned to IPE or placebo.

**Table 1:** Baseline characteristics of patients with metabolic syndrome (based on ≥3 risk factors) and without diabetes at baseline in REDUCE-IT

	Icosapent ethyl (*N* = 1453)	Placebo (*N* = 1413)	Overall (*N* = 2866)	*P*-value
Age (years)				0.64
Median (Q1–Q3)	62.0 (56.0–68.0)	63.0 (56.0–69.0)	62.0 (56.0–69.0)	
Age group, *n* (%)				0.43
<65 years	857 (59.0)	813 (57.5)	1670 (58.3)	
≥65 years	596 (41.0)	600 (42.5)	1196 (41.7)	
Sex, *n* (%)				0.68
Male	1169 (80.5)	1128 (79.8)	2297 (80.1)	
Female	284 (19.5)	285 (20.2)	569 (19.9)	
Race, *n* (%)				0.20
White	1366 (94.0)	1344 (95.1)	2710 (94.6)	
Black	11 (0.8)	15 (1.1)	26 (0.9)	
Asian	56 (3.9)	37 (2.6)	93 (3.2)	
CV risk category, *n* (%)				0.43
Secondary prevention cohort	1449 (99.7)	1411 (99.9)	2860 (99.8)	
Primary prevention cohort	4 (0.3)	2 (0.1)	6 (0.2)	
Waist circumference, *n* (%)				
Men ≥40 inches (102 cm)	769/1169 (65.8)	753/1128 (66.8)	1522/2297 (66.3)	0.78
Women ≥35 inches (88 cm)	242/284 (85.2)	258/285 (90.5)	500/569 (87.9)	0.06
Hypertension, *n* (%)				0.08
Yes	1174 (80.8)	1177 (83.3)	2351 (82.0)	
No	279 (19.2)	236 (16.7)	515 (18.0)	
Impaired fasting glucose metabolism, *n* (%)				0.11
Yes	819 (56.4)	839 (59.4)	1658 (57.9)	
No	633 (43.6)	574 (40.6)	1207 (42.1)	
Triglycerides, mg/dL				0.57
Median (Q1–Q3)	223.0 (183.0–281.0)	222.5 (183.5–278.5)	223.0 (183.5–279.5)	
LDL-C, mg/dL				0.27
Median (Q1–Q3)	76.0 (64.0–89.0)	77.0 (64.0–90.0)	77.0 (64.0–90.0)	
HDL-C, *n* (%)				
Men (<40 mg/dL)	684/1169 (58.5)	702/1128 (62.2)	1386/2297 (60.3)	0.08
Women (<50 mg/dL)	226/284 (79.6)	198/285 (69.5)	424/569 (74.5)	0.01
hsCRP (mg/L)				0.28
Median (Q1–Q3)	2.0 (1.0–4.0)	1.9 (1.0–3.8)	1.9 (1.0–3.9)	

Age (years) is at randomization.

**Table 2:** Baseline medications of patients with metabolic syndrome (based on ≥3 risk factors) and without diabetes at baseline in REDUCE-IT

Medication taken at baseline	Icosapent ethyl (*N* = 1453)	Placebo (*N* = 1413)	Overall (*N* = 2866)	*P*-value
	*n* (%)	*n* (%)	*n* (%)	
Antihypertensive	1402 (96.5)	1362 (96.4)	2764 (96.4)	0.89
Antiplatelet	1315 (90.5)	1267 (89.7)	2582 (90.1)	0.45
1 antiplatelet	900 (61.9)	876 (62.0)	1776 (62.0)	0.98
≥2 antiplatelets	415 (28.6)	391 (27.7)	806 (28.1)	0.60
Anticoagulant	140 (9.6)	120 (8.5)	260 (9.1)	0.29
Anticoagulant + antiplatelet	56 (3.9)	42 (3.0)	98 (3.4)	0.19
ACE inhibitor or ARB	1070 (73.6)	1074 (76.0)	2144 (74.8)	0.14
ACE	752 (51.8)	743 (52.6)	1495 (52.2)	0.66
ARB	328 (22.6)	343 (24.3)	671 (23.4)	0.28
Beta-blocker	1167 (80.3)	1127 (79.8)	2294 (80.0)	0.71
Statin	1450 (99.8)	1409 (99.7)	2859 (99.8)	0.68
Statin intensity				0.10
Low	39 (2.7)	58 (4.1)	97 (3.4)	
Moderate	889 (61.2)	863 (61.1)	1752 (61.1)	
High	522 (35.9)	488 (34.5)	1010 (35.2)	
Ezetimibe use	115 (7.9)	113 (8.0)	228 (8.0)	0.93

ACE, angiotensin-converting enzyme; ARB, angiotensin receptor blocker.

### Clinical outcomes

**Figure 1:**
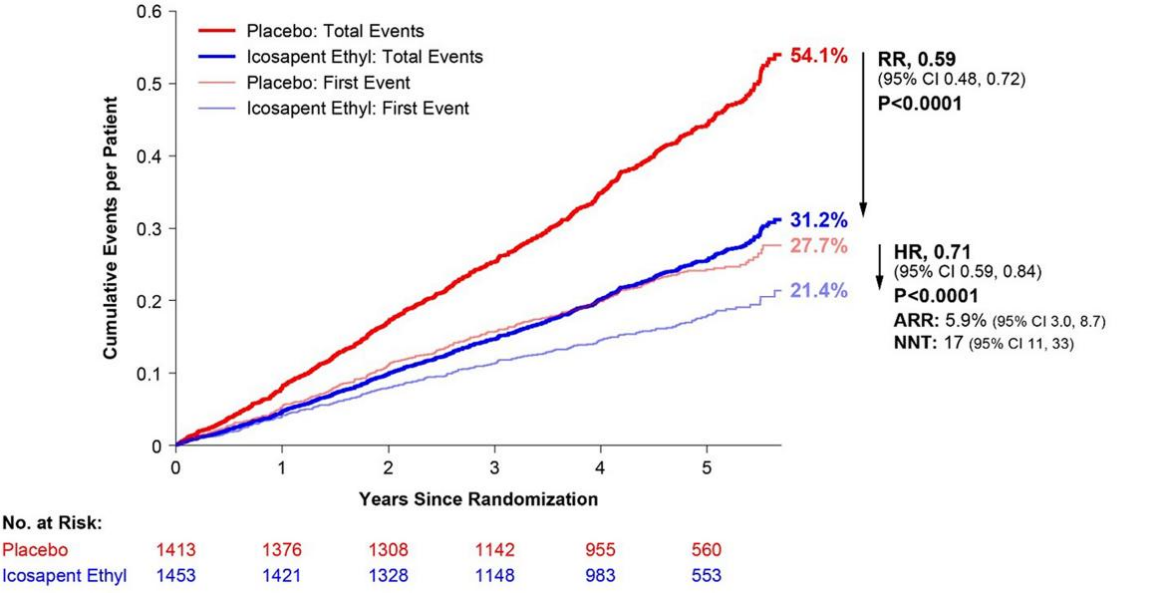
Total (first plus subsequent) and time to first primary composite endpoint in patients with metabolic syndrome*, but without diabetes. *Diagnosis of metabolic syndrome requires the presence of three or more of the following five risk factors using these baseline criteria: TG ≥ 150 mg/dL; low HDL-cholestrol <40 mg/dL if male and <50 mg/dL if female; fasting glucose ≥100 mg/dL OR on drug therapy for elevated glucose; blood pressure systolic ≥130 mmHg and/or diastolic ≥85 mmHg OR on antihypertensive therapy with medical history of hypertension; and a waist circumferences ≥35 inches (88 cm) for all women and Asian, Hispanic, or Latino men, and waist circumferences ≥40 inches (102 cm) for all other men.

Over a median follow-up time of 4.9 years, IPE-treated patients with MetSyn but without diabetes experienced a 29% relative risk reduction (RRR) for the time to first primary composite endpoint [HR: 0.71; 95% CI: 0.59–0.84; *P* < 0.0001; absolute risk reduction (ARR) = 5.9%; number needed to treat = 17] and a 41% reduction in total (first plus subsequent) events [rate ratio (RR): 0.59; (95% CI: 0.48–0.72); *P* < 0.0001], compared with placebo (*Figure 1*).

**Figure 2:**
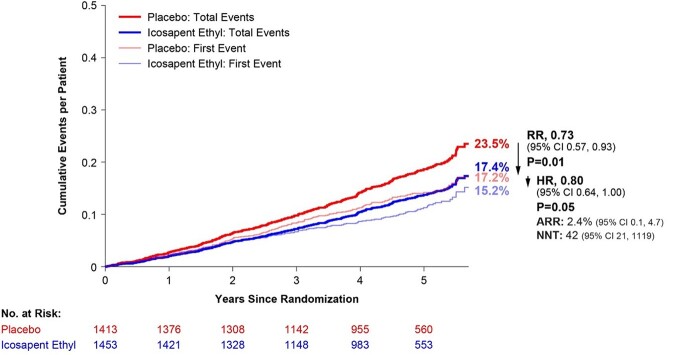
Total (first plus subsequent) and time to first key secondary composite endpoint in patients with metabolic syndrome*, but without diabetes. *Diagnosis of metabolic syndrome requires the presence of three or more of the following five risk factors using these baseline criteria: TG ≥ 150 mg/dL; low HDL-cholestrol <40 mg/dL if male and <50 mg/dL if female; fasting glucose ≥100 mg/dL OR on drug therapy for elevated glucose; blood pressure systolic ≥130 mmHg and/or diastolic ≥85 mmHg OR on antihypertensive therapy with medical history of hypertension; and a waist circumferences ≥35 inches (88 cm) for all women and Asian, Hispanic, or Latino men, and waist circumferences ≥40 inches (102 cm) for all other men.

Similarly, IPE use in patients with MetSyn who did not have diabetes at baseline was associated with a 20% reduction in the key secondary composite endpoint (HR: 0.80; 95% CI: 0.64–1.00; *P* = 0.05) and a 27% reduction in total events (RR: 0.73; 95% CI: 0.57–0.93; *P* = 0.01; *Figure 2*). No appreciable differences were observed in the primary or key secondary composite endpoint in IPE-treated patients with MetSyn who had pre-existing diabetes (see Supplementary material online, *Figure S1*).

Similarly, subgroup analysis for individual MetSyn components, except for high TGs, with IPE treatment effects on the primary endpoint is illustrated in Supplementary material online, *Figure S2*. Across these four MetSyn criteria, IPE significantly reduced the risk of primary endpoint events regardless of the presence or absence of each criterion in the ITT population with two exceptions—patients without elevated blood pressure and those without reduced HDL-C—although there was a non-statistically significant trend towards benefit with IPE (*P* = 0.07). Efficacy endpoints were further stratified in primary and secondary prevention patients with MetSyn (see Supplementary material online, *Figure S3*). Consistent with the main REDUCE-IT study results, IPE significantly reduced the risk of the primary and key secondary endpoints in the secondary prevention subgroup, with a trend towards benefit in the smaller primary prevention subgroup.

**Figure 3:**
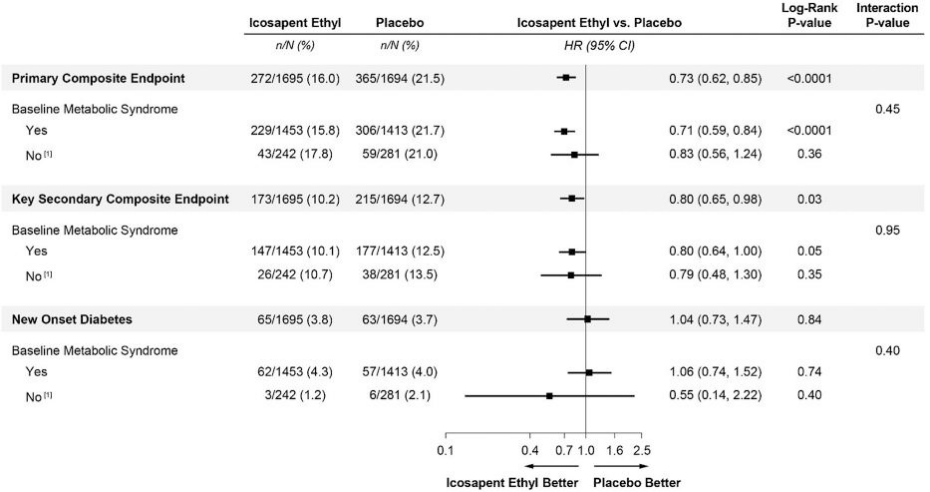
Primary, key secondary composite endpoints, and new-onset diabetes in patients without diabetes by baseline metabolic syndrome*. [1] Five hundred twenty-three subjects without diabetes did not meet the ≥3 criteria for metabolic syndrome diagnosis, including five icosapent ethyl and three placebo subjects with missing/unknown determination of metabolic syndrome. *Diagnosis of metabolic syndrome requires the presence of three or more of the following five risk factors using these baseline criteria: TG ≥ 150 mg/dL; low HDL-cholestrol <40 mg/dL if male and <50 mg/dL if female; fasting glucose ≥100 mg/dL OR on drug therapy for elevated glucose; blood pressure systolic ≥130 mmHg and/or diastolic ≥85 mmHg OR on antihypertensive therapy with medical history of hypertension; and a waist circumferences ≥35 inches (88 cm) for all women and Asian, Hispanic, or Latino men, and waist circumferences ≥40 inches (102 cm) for all other men.

A forest plot of primary and key secondary composite endpoints and development of new-onset diabetes based on treatment assignment and stratified by the presence or absence of MetSyn is shown in *Figure 3*. Icosapent ethyl use was associated with statistically significant reductions in CVD events only with MetSyn for patients without diabetes at baseline; however, there were no significant differences in primary or key secondary composite endpoints between the MetSyn groups (interaction *P*-values of 0.45 and 0.95, respectively). Treatment with IPE vs. placebo did not increase the risk of new-onset diabetes, irrespective of baseline MetSyn status (with MetSyn 4.3 vs. 4.0%, log-rank *P* = 0.74; and without MetSyn 1.2 vs. 2.1%, log-rank *P* = 0.40).

**Figure 4:**
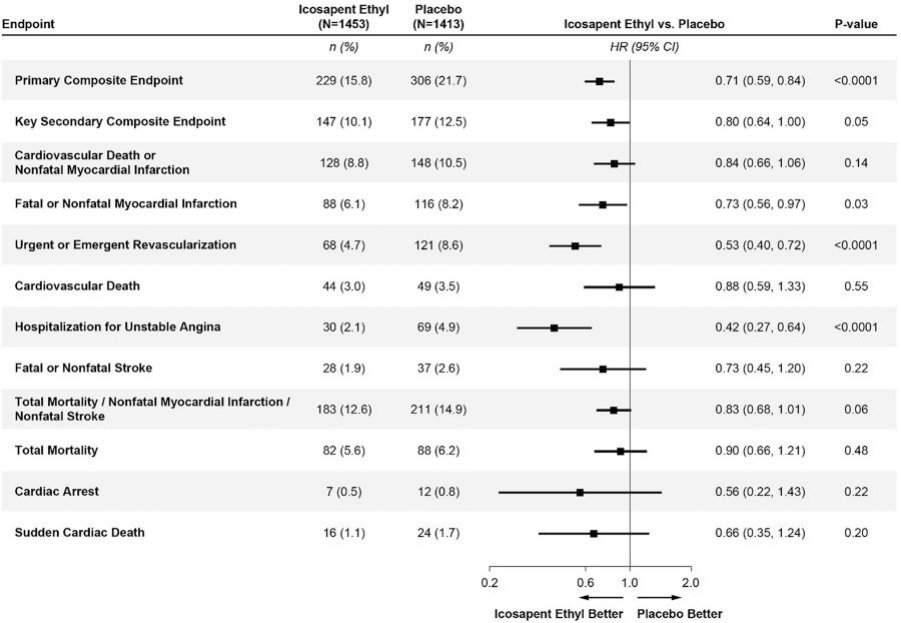
Efficacy endpoints in patients with metabolic syndrome*, but without diabetes at baseline. *Diagnosis of metabolic syndrome requires the presence of three or more of the following five risk factors using these baseline criteria TG ≥ 150 mg/dL; low HDL-cholestrol <40 mg/dL if male and <50 mg/dL if female; fasting glucose ≥100 mg/dL OR on drug therapy for elevated glucose; blood pressure systolic ≥130 mmHg and/or diastolic ≥85 mmHg OR on antihypertensive therapy with medical history of hypertension; and a waist circumferences ≥35 inches (88 cm) for all women and Asian, Hispanic, or Latino men, and waist circumferences ≥40 inches (102 cm) for all other men.

Further evaluation of efficacy endpoints in patients with MetSyn but without diabetes at baseline is illustrated in *Figure 4*. There was a 27% RRR in fatal/non-fatal MI (*P* = 0.03), 47% RRR in urgent/emergent revascularization (*P* < 0.0001), and 58% RRR in hospitalization for unstable angina (*P* < 0.0001), with associated ARRs of 2.1, 3.9, and 2.8%, respectively. Non-statistically significant reductions were observed in CV death or non-fatal MI (16%), fatal or non-fatal stroke (27%), the combination of total mortality, non-fatal MI and non-fatal stroke (17%), cardiac arrest (44%), and sudden cardiac death (34%).

Two medically significant adverse experiences associated with use of omega-3 fatty acids include atrial fibrillation/flutter and bleeding. In the subgroup of patients with MetSyn but without diabetes at baseline, hospitalization for positively adjudicated atrial fibrillation or flutter events was higher in participants assigned to IPE than placebo (2.9 vs. 1.8%; log-rank *P* = 0.06; Supplementary material online, *Figure S4*). Bleeding treatment-emergent adverse events (TEAEs) or haemorrhagic stroke endpoints trended higher with IPE vs. placebo (10.0 vs. 9.1%; Fisher’s exact *P* = 0.37), albeit with no significant differences for either bleeding TEAEs (9.8 vs. 8.9%; Fisher’s exact *P* = 0.41) or haemorrhagic stroke endpoints (0.2 vs. 0.3%; Fisher’s exact *P* = 0.72; Supplementary material online, *Figure S5*).

## Discussion

In this pre-specified analysis of REDUCE-IT, assignment to IPE with pre-existing MetSyn (based on ≥3 factors) but no history of diabetes at baseline resulted in significant reductions in the first event and also in total events (29 and 41%, respectively) of the primary composite endpoint compared with placebo; these results compared favourably with the overall REDUCE-IT results (25 and 30% reductions, respectively^7,9^). The risk of primary composite endpoint events in placebo-treated patients with MetSyn aligns with the overall REDUCE-IT placebo cohort (21.7 vs. 22.0%^7^) over the median 4.9-year follow-up period.

Even in the absence of diabetes at baseline, these data are consistent with the notion that secondary prevention patients with MetSyn pose higher CVD risk than comparably risk-defined patients without MetSyn,^2,10^ and reflects in part, an atherothrombotic milieu characterized by chronic inflammation, oxidative stress and endothelial dysfunction.^11,12^ That IPE appreciably offsets the excessive risk inherent in CVD patients with MetSyn is supported by investigational studies demonstrating the anti-inflammatory, antithrombotic, and membrane stabilizing effects of highly purified EPA.^13^ In contrast, other therapies added to statins have failed to effectively lower CVD risk in high-risk patients with MetSyn or with at least two MetSyn criteria (e.g. low HDL-C and high TG).^14–17^

As the prevalence of MetSyn continues to rise beyond the approximate one in four adults currently afflicted worldwide, owing in part to the growing epidemic of obesity and diabetes,^18^ treatment considerations include lifestyle interventions, notably weight reduction and/or improvement in associated cardiometabolic factors identified in small-scaled trials.^19–22^ We await the initiation of larger randomized clinical trials that specifically address these measures, although the Semaglutide Effects on Heart Disease and Stroke in Patients with Overweight or Obesity (SELECT) trial^23^ may provide insight on the effectiveness of this GLP-1 receptor agonist in the cohort with MetSyn but without diabetes at baseline. In the meantime, IPE represents one of only a handful of therapies^24,25^ that may effectively reduce CVD risk in MetSyn patients without exhibiting robust effects on other MetSyn parameters.

### Study limitations

Limitations of this pre-specified analysis were its exploratory nature and relatively small number of events in certain subgroups or for certain endpoints, including cardiac arrest and sudden cardiac death. Nevertheless, the trends observed were consistent with the favourable results identified in the primary and key secondary composite endpoints. In addition, variation in subjective measures (e.g. waist circumference) may have affected the classification of MetSyn.

## Conclusions

In this pre-specified analysis of patients with MetSyn from REDUCE-IT, patients with MetSyn but without diabetes at baseline who were assigned to IPE treatment experienced significantly fewer first and total events compared with placebo. These data expand the growing list of benefits attributable to IPE, including prior MI, percutaneous coronary intervention or coronary bypass grafting, chronic kidney disease, heart failure, and history of cigarette smoking.^26–31^ Taken together, IPE is effective in a variety of clinical settings including patients with MetSyn at high CV risk.

## Lead author biography


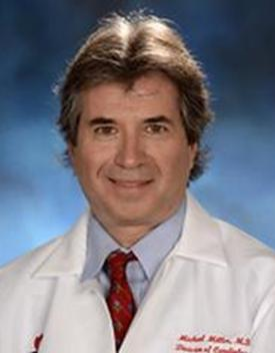
Michael Miller, MD, is a cardiologist and Professor of Medicine at the Hospital of the University of Pennsylvania and Chief of Medicine at the Corporal Michael Crescenz Veterans Affairs Medical Center, Philadelphia, PA, USA. Dr Miller has had a longstanding interest in triglyceride metabolism. He and his collaborators were the first to show optimal fasting triglyceride levels to be <100 mg/dL (1.13 mmol/L) and higher levels [e.g. >150 mg/dL (1.7 mmol/L)] to predict recurrent cardiovascular events despite low on-treatment LDL-C [<70 mg/dL (1.8 mmol/L)]. His studies on fatty acids, including EPA, have spanned multiple decades.

## Supplementary Material

oead114_Supplementary_Data

## Data Availability

The data underlying this article will be shared on reasonable request to the corresponding author.

## References

[1] Hirode G , Wong RJ. Trends in the prevalence of metabolic syndrome in the United States, 2011–2016. JAMA 2020;323:2526–2528. 32573660 10.1001/jama.2020.4501 PMC7312413

[2] Alberti KG , Eckel RH, Grundy SM, Zimmet PZ, Cleeman JI, Donato KA, Fruchart JC, James WP, Loria CM, Smith SC Jr; International Diabetes Federation Task Force on Epidemiology and Prevention; National Heart, Lung, and Blood Institute; American Heart Association; World Heart Federation; International Atherosclerosis Society; International Association for the Study of Obesity. Harmonizing the metabolic syndrome: a joint interim statement of the International Diabetes Federation Task Force on Epidemiology and Prevention; National Heart, Lung, and Blood Institute; American Heart Association; World Heart Federation; International Atherosclerosis Society; and International Association for the Study of Obesity. Circulation 2009;120:1640–1645. 19805654 10.1161/CIRCULATIONAHA.109.192644

[3] Mottillo S , Filion KB, Genest J, Joseph L, Pilote L, Poirier P, Rinfret S, Schiffrin EL, Eisenberg MJ. The metabolic syndrome and cardiovascular risk a systematic review and meta-analysis. J Am Coll Cardiol 2010;56:1113–1132. 20863953 10.1016/j.jacc.2010.05.034

[4] Burger PM , Koudstaal S, Dorresteijn JAN, Savarese G, van der Meer MG, de Borst GJ, Mosterd A, Visseren FLJ; UCC-SMART Study Group. Metabolic syndrome and risk of incident heart failure in non-diabetic patients with established cardiovascular disease. Int J Cardiol 2023;379:66–75. 36907452 10.1016/j.ijcard.2023.03.024

[5] Li X , Liang Q, Zhong J, Gan L, Zuo L. The effect of metabolic syndrome and its individual components on renal function: a meta-analysis. J Clin Med 2023;12:1614. 36836149 10.3390/jcm12041614 PMC9962508

[6] Angelico F , Baratta F, Coronati M, Ferro D, Del Ben M. Diet and metabolic syndrome: a narrative review. Intern Emerg Med 2023;18:1007–1017. 36929350 10.1007/s11739-023-03226-7

[7] Bhatt DL , Steg PG, Miller M, Brinton EA, Jacobson TA, Ketchum SB, Doyle RT Jr, Juliano RA, Jiao L, Granowitz C, Tardif JC, Ballantyne CM; REDUCE-IT Investigators. Cardiovascular risk reduction with icosapent ethyl for hypertriglyceridemia. N Engl J Med 2019;380:11–22. 30415628 10.1056/NEJMoa1812792

[8] Bhatt DL , Steg PG, Brinton EA, Jacobson TA, Miller M, Tardif JC, Ketchum SB, Doyle RT Jr, Murphy SA, Soni PN, Braeckman RA, Juliano RA, Ballantyne CM; REDUCE-IT Investigators. Rationale and design of REDUCE-IT: reduction of cardiovascular events with icosapent ethyl-intervention trial. Clin Cardiol 2017;40:138–148. 28294373 10.1002/clc.22692 PMC5396348

[9] Bhatt DL , Steg PG, Miller M, Brinton EA, Jacobson TA, Ketchum SB, Doyle RT Jr, Juliano RA, Jiao L, Granowitz C, Tardif JC, Gregson J, Pocock SJ, Ballantyne CM; REDUCE-IT Investigators. Effects of icosapent ethyl on total ischemic events: from REDUCE-IT. J Am Coll Cardiol 2019;73:2791–2802. 30898607 10.1016/j.jacc.2019.02.032

[10] Guembe MJ , Fernandez-Lazaro CI, Sayon-Orea C, Toledo E, Moreno-Iribas C; RIVANA Study Investigators. Risk for cardiovascular disease associated with metabolic syndrome and its components: a 13-year prospective study in the RIVANA cohort. Cardiovasc Diabetol 2020;19:195. 33222691 10.1186/s12933-020-01166-6 PMC7680587

[11] Silveira Rossi JL , Barbalho SM, Reverete de Araujo R, Bechara MD, Sloan KP, Sloan LA. Metabolic syndrome and cardiovascular diseases: going beyond traditional risk factors. Diabetes Metab Res Rev 2022;38:e3502. 34614543 10.1002/dmrr.3502

[12] Grandl G , Wolfrum C. Hemostasis, endothelial stress, inflammation, and the metabolic syndrome. Semin Immunopathol 2018;40:215–224. 29209827 10.1007/s00281-017-0666-5 PMC5809518

[13] Mason RP , Libby P, Bhatt DL. Emerging mechanisms of cardiovascular protection for the omega-3 fatty acid eicosapentaenoic acid. Arterioscler Thromb Vasc Biol 2020;40:1135–1147. 32212849 10.1161/ATVBAHA.119.313286 PMC7176343

[14] AIM-HIGH Investigators; Boden WE, Probstfield JL, Anderson T, Chaitman BR, Desvignes-Nickens P, Koprowicz K, McBride R, Teo K, Weintraub W. Niacin in patients with low HDL cholesterol levels receiving intensive statin therapy. N Engl J Med 2011;365:2255–2267. 22085343 10.1056/NEJMoa1107579

[15] Lyubarova R , Robinson JG, Miller M, Simmons DL, Xu P, Abramson BL, Elam MB, Brown TM, McBride R, Fleg JL, Desvigne-Nickens P, Ayenew W, Boden WE; Atherothrombosis Intervention in Metabolic Syndrome with Low HDL/High Triglycerides and Impact on Global Health Outcomes (AIM-HIGH) Investigators. Metabolic syndrome cluster does not provide incremental prognostic information in patients with stable cardiovascular disease: a post hoc analysis of the AIM-HIGH trial. J Clin Lipidol 2017;11:1201–1211 28807460 10.1016/j.jacl.2017.06.017 PMC5612889

[16] Nicholls SJ , Lincoff AM, Garcia M, Bash D, Ballantyne CM, Barter PJ, Davidson MH, Kastelein JJP, Koenig W, McGuire DK, Mozaffarian D, Ridker PM, Ray KK, Katona BG, Himmelmann A, Loss LE, Rensfeldt M, Lundström T, Agrawal R, Menon V, Wolski K, Nissen SE. Effect of high-dose omega-3 fatty acids vs corn oil on major adverse cardiovascular events in patients at high cardiovascular risk: the STRENGTH randomized clinical trial. JAMA 2020;324:2268–2280. 33190147 10.1001/jama.2020.22258 PMC7667577

[17] Das Pradhan A , Glynn RJ, Fruchart JC, MacFadyen JG, Zaharris ES, Everett BM, Campbell SE, Oshima R, Amarenco P, Blom DJ, Brinton EA, Eckel RH, Elam MB, Felicio JS, Ginsberg HN, Goudev A, Ishibashi S, Joseph J, Kodama T, Koenig W, Leiter LA, Lorenzatti AJ, Mankovsky B, Marx N, Nordestgaard BG, Páll D, Ray KK, Santos RD, Soran H, Susekov A, Tendera M, Yokote K, Paynter NP, Buring JE, Libby P, Ridker PM; PROMINENT Investigators. Triglyceride lowering with pemafibrate to reduce cardiovascular risk. N Engl J Med 2022;387:1923–1934. 36342113 10.1056/NEJMoa2210645

[18] O’Neill S , O’Driscoll L. Metabolic syndrome: a closer look at the growing epidemic and its associated pathologies. Obes Rev 2015;16:1–12. 10.1111/obr.12229 25407540

[19] Miller M , Stone NJ, Ballantyne C, Bittner V, Criqui MH, Ginsberg HN, Goldberg AC, Howard WJ, Jacobson MS, Kris-Etherton PM, Lennie TA, Levi M, Mazzone T, Pennathur S; American Heart Association Clinical Lipidology, Thrombosis, and Prevention Committee of the Council on Nutrition, Physical Activity, and Metabolism; Council on Arteriosclerosis, Thrombosis and Vascular Biology; Council on Cardiovascular Nursing; Council on the Kidney in Cardiovascular Disease. Triglycerides and cardiovascular disease: a scientific statement from the American Heart Association. Circulation 2011;123:2292–2333. 21502576 10.1161/CIR.0b013e3182160726

[20] Shin JY , Kim JY, Kang HT, Han KH, Shim JY. Effect of fruits and vegetables on metabolic syndrome: a systematic review and meta-analysis of randomized controlled trials. Int J Food Sci Nutr 2015;66:416–425. 25945735 10.3109/09637486.2015.1025716

[21] Chu P , Gotink RA, Yeh GY, Goldie SJ, Hunink MG. The effectiveness of yoga in modifying risk factors for cardiovascular disease and metabolic syndrome: a systematic review and meta-analysis of randomized controlled trials. Eur J Prev Cardiol 2016;23:291–307. 25510863 10.1177/2047487314562741

[22] Miller M , Sorkin JD, Mastella L, Sutherland A, Rhyne J, Donnelly P, Simpson K, Goldberg AD. Poly is more effective than mono—unsaturated fat for dietary management in the metabolic syndrome: the MUFFIN study. J Clin Lipidol 2016;10:996–1003. 27578132 10.1016/j.jacl.2016.04.011 PMC5010036

[23] Ryan DH , Lingvay I, Colhoun HM, Deanfield J, Emerson SS, Kahn SE, Kushner RF, Marso S, Plutzky J, Brown-Frandsen K, Gronning MOL, Hovingh GK, Holst AG, Ravn H, Lincoff AM. Semaglutide effects on cardiovascular outcomes in people with overweight or obesity (SELECT) rationale and design. Am Heart J 2020;229:61–69. 32916609 10.1016/j.ahj.2020.07.008

[24] Deedwania P , Barter P, Carmena R, Fruchart JC, Grundy SM, Haffner S, Kastelein JJ, LaRosa JC, Schachner H, Shepherd J, Waters DD; Treating to New Targets Investigators. Reduction of low-density lipoprotein cholesterol in patients with coronary heart disease and metabolic syndrome: analysis of the treating to new targets study. Lancet 2006;368:919–928. 16962881 10.1016/S0140-6736(06)69292-1

[25] Ostadal P , Steg PG, Poulouin Y, Bhatt DL, Bittner VA, Chua T, Diaz R, Goodman SG, Huo Y, Jukema JW, Karpov Y, Pordy R, Scemama M, Szarek M, White HD, Schwartz GG; ODYSSEY OUTCOMES Investigators. Metabolic risk factors and effect of alirocumab on cardiovascular events after acute coronary syndrome: a post-hoc analysis of the ODYSSEY OUTCOMES randomised controlled trial. Lancet Diabetes Endocrinol. 2022;10:330–340. 35378068 10.1016/S2213-8587(22)00043-2

[26] Gaba P , Bhatt DL, Steg PG, Miller M, Brinton EA, Jacobson TA, Ketchum SB, Juliano RA, Jiao L, Doyle RT Jr, Granowitz C, Tardif JC, Giugliano RP, Martens FMAC, Gibson CM, Ballantyne CM; REDUCE-IT Investigators. Prevention of cardiovascular events and mortality with icosapent ethyl in patients with prior myocardial infarction. J Am Coll Cardiol 2022;79:1660–1671. 35483753 10.1016/j.jacc.2022.02.035

[27] Peterson BE , Bhatt DL, Steg PG, Miller M, Brinton EA, Jacobson TA, Ketchum SB, Juliano RA, Jiao L, Doyle RT Jr, Granowitz C, Gibson CM, Pinto D, Giugliano RP, Budoff MJ, Tardif JC, Verma S, Ballantyne CM; REDUCE-IT Investigators. Treatment with icosapent ethyl to reduce ischemic events in patients with prior percutaneous coronary intervention: insights from REDUCE-IT PCI. J Am Heart Assoc 2022;11:e022937 35261279 10.1161/JAHA.121.022937 PMC9075300

[28] Verma S , Bhatt DL, Steg PG, Miller M, Brinton EA, Jacobson TA, Dhingra NK, Ketchum SB, Juliano RA, Jiao L, Doyle RT Jr, Granowitz C, Gibson CM, Pinto D, Giugliano RP, Budoff MJ, Mason RP, Tardif JC, Ballantyne CM; REDUCE-IT Investigators. Icosapent ethyl reduces ischemic events in patients with a history of previous coronary artery bypass grafting: REDUCE-IT CABG. Circulation 2021;144:1845–1855. 34710343 10.1161/CIRCULATIONAHA.121.056290

[29] Majithia A , Bhatt DL, Friedman AN, Miller M, Steg PG, Brinton EA, Jacobson TA, Ketchum SB, Juliano RA, Jiao L, Doyle RT Jr, Granowitz C, Budoff M, Preston Mason R, Tardif JC, Boden WE, Ballantyne CM. Benefits of icosapent ethyl across the range of kidney function in patients with established cardiovascular disease or diabetes: REDUCE-IT RENAL. Circulation 2021;144:1750–1759. 34706555 10.1161/CIRCULATIONAHA.121.055560 PMC8614567

[30] Selvaraj S , Bhatt DL, Steg PG, Miller M, Brinton EA, Jacobson TA, Juliano RA, Jiao L, Tardif JC, Ballantyne CM; REDUCE-IT Investigators. Impact of icosapent ethyl on cardiovascular risk reduction in patients with heart failure in REDUCE-IT. J Am Heart Assoc 2022;11:e024999. 35377160 10.1161/JAHA.121.024999 PMC9075460

[31] Miller M , Bhatt DL, Steg PG, Brinton EA, Jacobson TA, Jiao L, Tardif JC, Ballantyne CM, Budoff M, Mason RP. Potential effects of icosapent ethyl on cardiovascular outcomes in cigarette smokers: REDUCE-IT smoking. Eur Heart J Cardiovasc Pharmacother 2023;9:129–137. 35953437 10.1093/ehjcvp/pvac045 PMC9892866

