# Correction to: Effectiveness of icosapent ethyl on first and total cardiovascular events in patients with metabolic syndrome, but without diabetes: REDUCE-IT MetSyn

**DOI:** 10.1093/ehjopen/oeae004

**Published:** 2024-03-01

This is a correction to: Michael Miller, Deepak L Bhatt, Eliot A Brinton, Terry A Jacobson, Philippe Gabriel Steg, Armando Lira Pineda, Steven B Ketchum, Ralph T Doyle, Jean-Claude Tardif, Christie M Ballantyne, on behalf of the REDUCE-IT Investigators, Effectiveness of icosapent ethyl on first and total cardiovascular events in patients with metabolic syndrome, but without diabetes: REDUCE-IT MetSyn, *European Heart Journal Open*, Volume 3, Issue 6, November 2023, oead114, https://doi.org/10.1093/ehjopen/oead114

Several corrections have been made to the originally published version of this manuscript.

In the graphical abstract, “Diabetes” has been added to the list of Keywords.

In the Introduction, “(iii) glucose ≥100 mg/dL” has been corrected to read “(iii) fasting glucose ≥100 mg/dL”.

In the following sentence, the word “patents” has been corrected to read “patients”:

However, evaluation of IPE in patents with MetSyn was not a primary analysis in the REDUCE-IT study.

The following sentence has been corrected to include the phrase “of Metsyn as a pre-specified subgroup analysis in REDUCE-IT”:

Consequently, the rationale for the current study, as a pre-specified subgroup analysis in REDUCE-IT, was two-fold: (i) examine the extent to which IPE may be beneficial in patients with MetSyn who did not have diabetes at baseline and (ii) determine whether IPE had any impact on the risk of new-onset diabetes in patients with MetSyn.

The following corrections have been made to Table 1:

“Impaired glucose metabolism” has been corrected to read “Impaired fasting glucose metabolism”.

Information on triglycerides has been added to the Table.

In “HDL-C, mg/dL, n (%)”, mg/dL has been deleted.

In Table 2, in the Beta-blocker row, 1127 (80.0) has been corrected to 1127 (79.8).

In Conclusions, the beginning of the following sentence has been corrected to read “In this pre-specified analysis of patients with MetSyn from REDUCE-IT”:

In this pre-specified analysis from REDUCE-IT, patients with MetSyn but without diabetes at baseline who were assigned to IPE treatment experienced significantly fewer first and total events compared with placebo.

The publication year in reference 3 has been corrected from 2020 to 2010

