# Supplementary material for: Effectiveness of icosapent ethyl on first and total cardiovascular events in patients with metabolic syndrome, but without diabetes: REDUCE-IT MetSyn
Source: Eur Heart J Open. 2023 Nov 12;3(6):oead114. doi: 10.1093/ehjopen/oead114 (PMC10684296; doi:10.1093/ehjopen/oead114)
Supplement: oead114_Supplementary_Data [file oead114_supplementary_data.docx]

**SUPPLEMENTARY MATERIAL**

**Effectiveness of Icosapent Ethyl on First and Total Cardiovascular Events in Patients with Metabolic Syndrome, but without Diabetes: REDUCE-IT MetSyn**

Michael Miller, M.D., Deepak L. Bhatt, M.D., M.P.H., Eliot A. Brinton, M.D., Terry A. Jacobson, M.D., Ph. Gabriel Steg, M.D., Armando Lira Pineda, M.D., Steven B. Ketchum, Ph.D., Ralph T. Doyle, Jr., B.A., Jean-Claude Tardif, M.D., Christie M. Ballantyne, M.D., on Behalf of the REDUCE-IT Investigators

**Table of Contents**

**Supplementary Figure 1.** Primary and Key Secondary Composite Efficacy Endpoints

in Patients with Metabolic Syndrome by Diabetes Status at Baseline, Intention-to-Treat (ITT) Population…………………………………………………………………………………………………2

**Supplementary Figure 2.** Primary Composite Efficacy Endpoint by Metabolic Syndrome Risk Factors at Baseline, Intention-to-Treat (ITT) Population………………..……………………………3

**Supplementary Figure 3.** Primary and Key Secondary Composite Efficacy Endpoints in Patients With Metabolic Syndrome (based on ≥3 risk factors) by CV Risk Category, Intention-to-Treat (ITT) Population……………………………………………………………………………………4

**Supplementary Figure 4.** Treatment-Emergent Atrial Fibrillation and Atrial Flutter Events in Patients With Metabolic Syndrome (based on ≥3 risk factors), but Without Diabetes at Baseline…………….……………………………………………………………………………………...5

**Supplementary Figure 5.** Treatment-Emergent Bleeding Adverse Events or Hemorrhagic Stroke in Patients With Metabolic Syndrome (based on ≥3 risk factors), but Without Diabetes at Baseline……………………………………………………………………………………………………6

**Supplementary Figure 1.** Primary and Key Secondary Composite Efficacy Endpoints in Patients with Metabolic Syndrome* by Diabetes Status at Baseline, Intention-to-Treat (ITT) Population

**
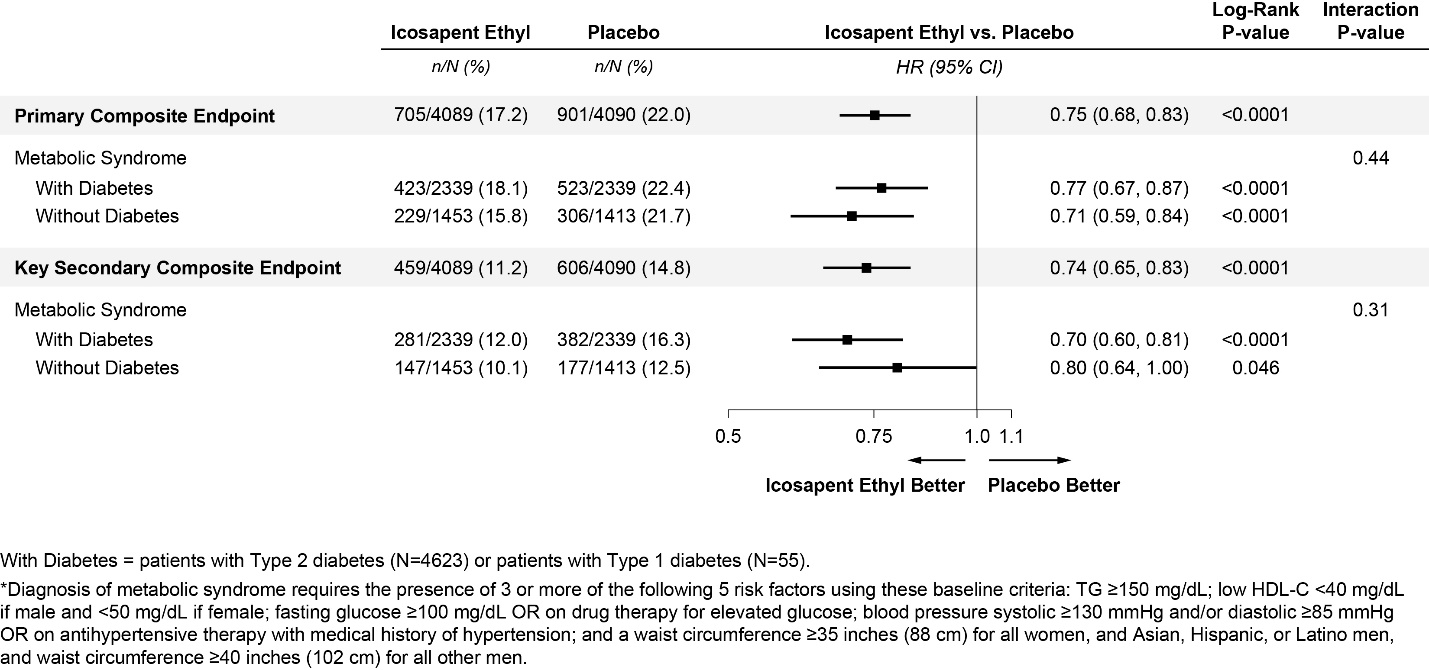
**

**Supplementary Figure 2.** Primary Composite Efficacy Endpoint by Metabolic Syndrome Risk Factors at Baseline, Intention-to-Treat (ITT) Population

**
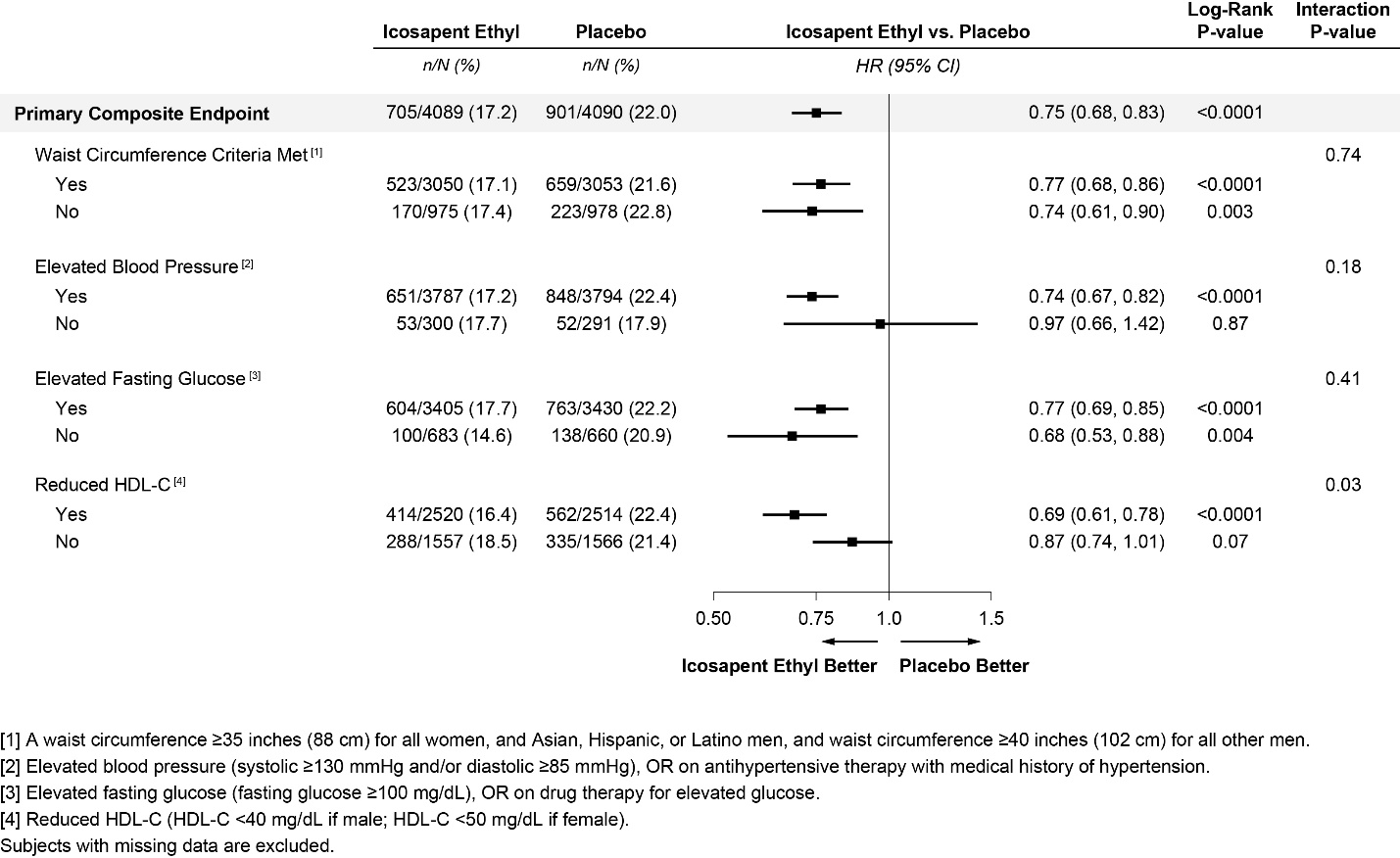
**

**Supplementary Figure 3.** Primary and Key Secondary Composite Efficacy Endpoints in Patients With Metabolic Syndrome (based on ≥3 risk factors) by CV Risk Category, Intention-to-Treat (ITT) Population

**
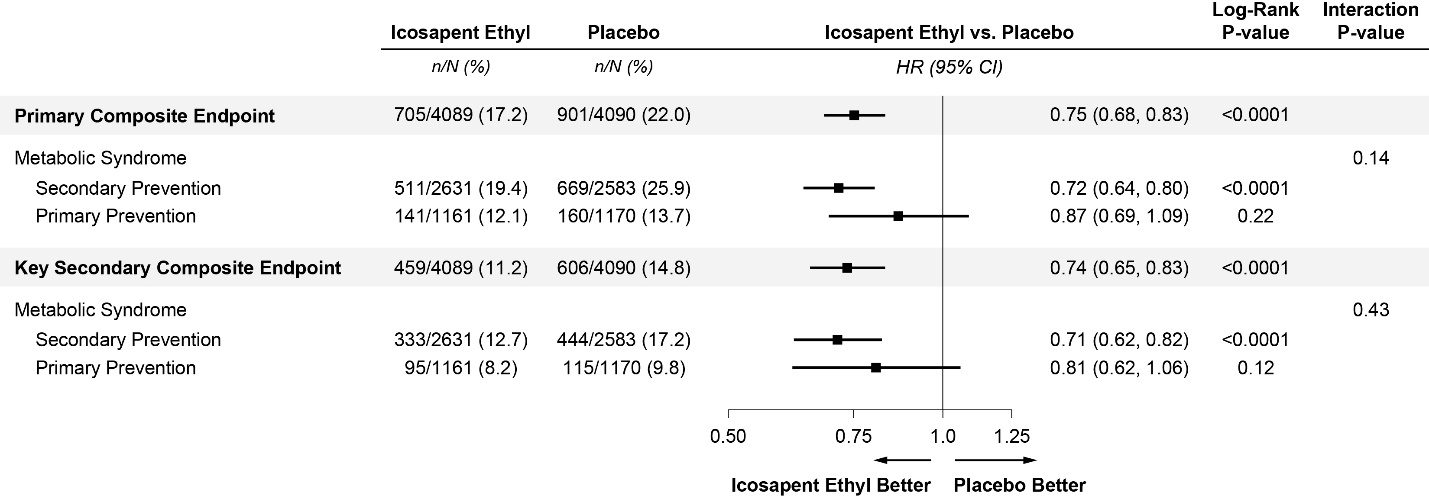
**

**Supplementary Figure 4.** Treatment-Emergent Atrial Fibrillation and Atrial Flutter Events in Patients With Metabolic Syndrome (based on ≥3 risk factors), but Without Diabetes at Baseline

**
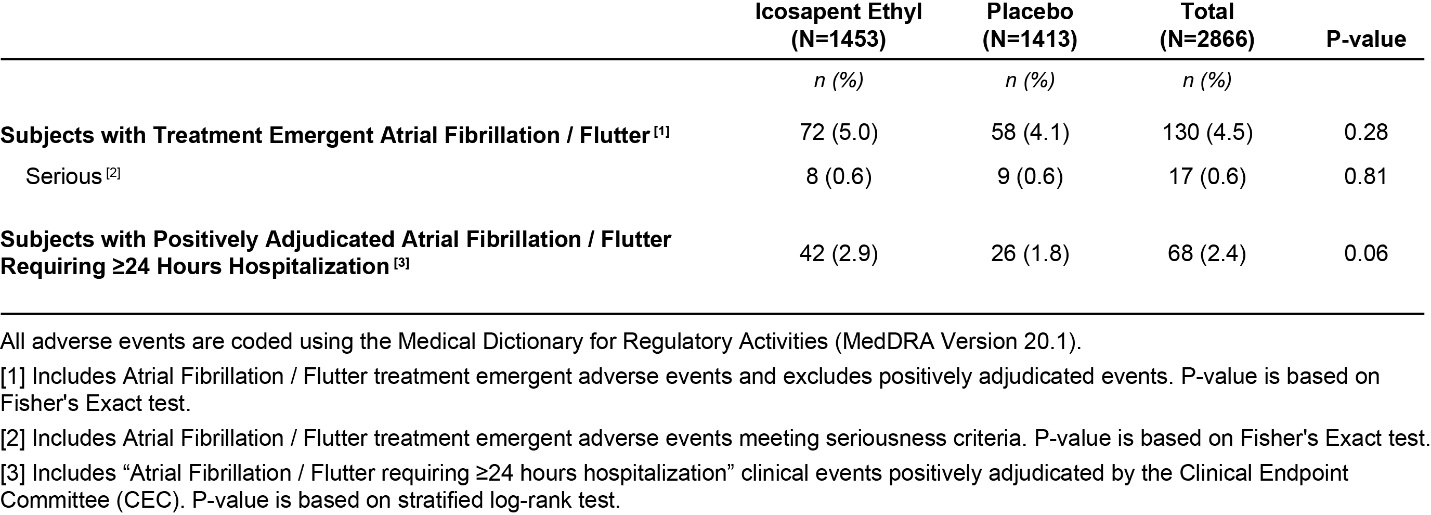
**

**Supplementary Figure 5.** Treatment-Emergent Bleeding Adverse Events or Hemorrhagic Stroke in Patients With Metabolic Syndrome (based on ≥3 risk factors), but Without Diabetes at Baseline

**
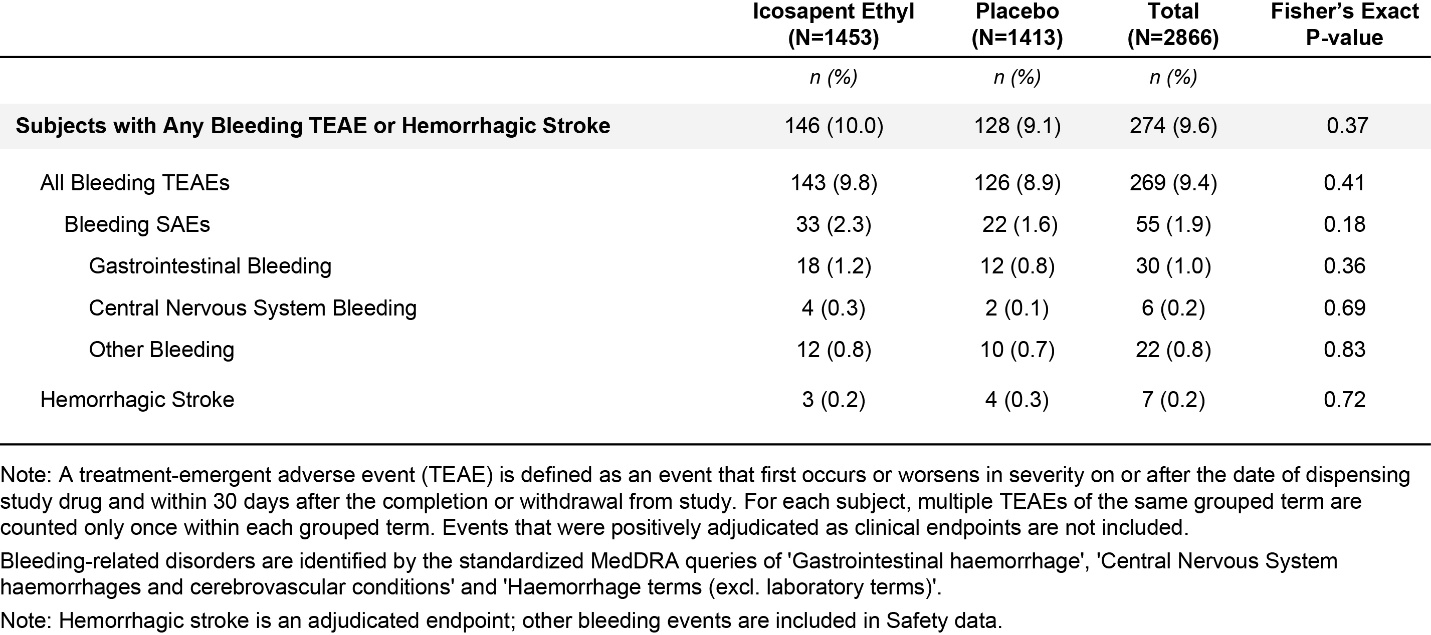
**
